# Negative circular polarization emissions from WSe_2_/MoSe_2_ commensurate heterobilayers

**DOI:** 10.1038/s41467-018-03869-7

**Published:** 2018-04-10

**Authors:** Wei-Ting Hsu, Li-Syuan Lu, Po-Hsun Wu, Ming-Hao Lee, Peng-Jen Chen, Pei-Ying Wu, Yi-Chia Chou, Horng-Tay Jeng, Lain-Jong Li, Ming-Wen Chu, Wen-Hao Chang

**Affiliations:** 1 Department of Electrophysics, National Chiao Tung University, Hsinchu, 30010 Taiwan; 2 Center for Condensed Matter Sciences, National Taiwan University, Taipei, 10617 Taiwan; 3 Institute of Physics, Academia Sinica, Taipei, 11529 Taiwan; 4 Department of Physics, National Tsing Hua University, Hsinchu, 30013 Taiwan; 5 Physical Sciences and Engineering, King Abdullah University of Science and Technology, Thuwal, 23955-6900 Saudi Arabia; 6 Center for Emergent Functional Matter Science, National Chiao Tung University, Hsinchu, 30010 Taiwan

## Abstract

Van der Waals heterobilayers of transition metal dichalcogenides with spin–valley coupling of carriers in different layers have emerged as a new platform for exploring spin/valleytronic applications. The interlayer coupling was predicted to exhibit subtle changes with the interlayer atomic registry. Manually stacked heterobilayers, however, are incommensurate with the inevitable interlayer twist and/or lattice mismatch, where the properties associated with atomic registry are difficult to access by optical means. Here, we unveil the distinct polarization properties of valley-specific interlayer excitons using epitaxially grown, commensurate WSe_2_/MoSe_2_ heterobilayers with well-defined (AA and AB) atomic registry. We observe circularly polarized photoluminescence from interlayer excitons, but with a helicity opposite to the optical excitation. The negative circular polarization arises from the quantum interference imposed by interlayer atomic registry, giving rise to distinct polarization selection rules for interlayer excitons. Using selective excitation schemes, we demonstrate the optical addressability for interlayer excitons with different valley configurations and polarization helicities.

## Abstract (web summary)

The interlayer coupling in van der Waals heterostructures is sensitive to the interlayer atomic registry. Here, the authors investigate the polarisation properties of epitaxially grown, commensurate WSe_2_/MoSe_2_ heterobilayers with well-defined atomic registry, and observe negative, circularly polarized photoluminescence from interlayer excitons.

## Introduction

Excitons in monolayer semiconductors, such as transition metal dichalcogenides (TMDs), are endowed with spin and valley degrees of freedom^1–5^. Recent advances in van der Waals heterojunctions formed by vertical stacking of different TMD monolayers further enable the generation of carriers in different layers with enriched valley configurations for exploring new spin/valleytronic applications^6–8^. Van der Waals heterobilayers (hBLs) of TMDs feature type-II band alignment, which can separate photoexcited electrons and holes into different layers through ultrafast charge transfer^9–13^ and can host long-lived interlayer excitons due to their spatially indirect nature^6–8^. Inheriting from the coupled spin–valley physics in the constituent monolayers, TMD hBLs further enrich the interplay of internal degrees of freedom, including the spin and valley pseudospin of electrons and holes confined in different monolayers^3^. In TMD homobilayers, the interplay of spin and valley pseudospins can lead to numerous exotic phenomena, such as the spin–layer locking effect^14^, the magnetoelectric effect^15^, and the electric-field-induced Zeeman-type splitting^16^. Recently, valley-specific interlayer excitons with long valley lifetimes have been realized in manually stacked WSe_2_/MoSe_2_ hBLs^7^. The valley-specific interlayer excitons were found to emit circularly polarized photoluminescence (PL) that retains the helicity of optical excitations. Theory has predicted that the valley optical selection rules and dipole strength of interlayer excitons are sensitive to the interlayer atomic registry^17, 18^. However, manually stacked hBLs are generally incommensurate with inevitable interlayer twist and/or lattice mismatch, which could lead to a periodic variation of the atomic registry between individual monolayers, i.e., the so-called Moiré superlattice^19, 20^. Conventional optical measurements are unable to resolve such variations in the atomic registry even for a very small twist angle (*θ*≈0.5°), where the Moiré periodicity (~40 nm) of hBLs can exceed the exciton radius (~1–2 nm), but remains much smaller than the typical spatial resolution (~1 μm) of optical measurements. Recently, periodical modulations in the local bandgap of rotationally aligned MoS_2_/WSe_2_ hBLs have been resolved by scanning tunneling microscopy and spectroscopy^21^. However, the experimental connection between the valley optical selection rules of interlayer excitons and the interlayer atomic registry of hBLs is still missing thus far.

Here we address how the interlayer atomic registry impacts the optical transition and polarization properties of interlayer excitons by using commensurate WSe_2_/MoSe_2_ hBLs formed by direct growth using chemical vapor deposition (CVD). The commensurate interlayer stacking with rotational alignment and long-range order facilitates us to unveil the valley optical selection rule of interlayer excitons in TMD hBLs. We observe circularly polarized photoluminescence (PL) from interlayer excitons, but with a helicity opposite to the optical excitation. The negative circular polarization arises from the quantum interference imposed by interlayer atomic registry, giving rise to distinct polarization selection rules for interlayer excitons. The impacts of stacking order on the formation processes of bright interlayer exciton states with different valley configurations are discussed. Selective excitations at different monolayers further demonstrate the optical addressability of interlayer excitons with different valley configurations and polarization helicities, providing a new scheme for exploring spin/valleytronic applications based on van der Waals heterostructures.

## Results

### Epitaxially grown commensurate WSe_2_/MoSe_2_ heterobilayers

**Fig. 1:**
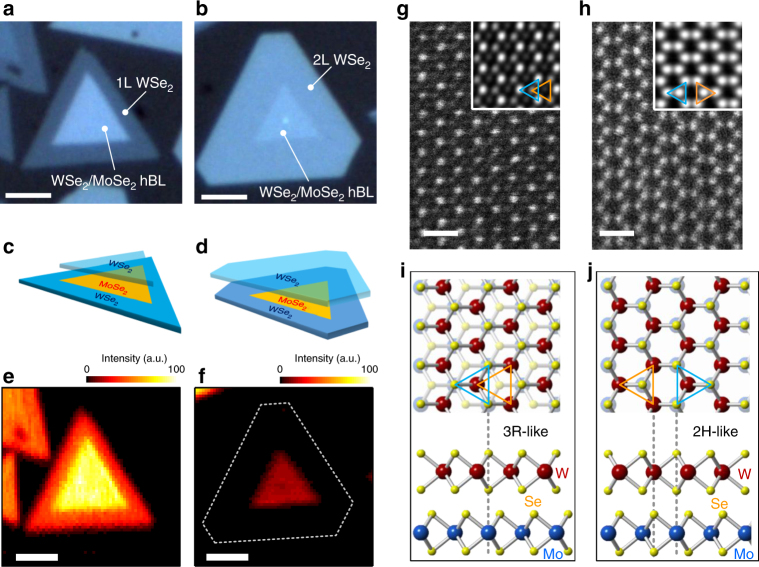
WSe_2_/MoSe_2_ hBLs with AA and AB stacking. **a**, **b** Optical images of hBLs with AA (**a**) and AB (**b**) stacking. The scale bar is 3 μm. **c**, **d** Schematics of heterostructures shown in **a** and **b**, respectively. **e**, **f** SHG intensity mapping for the hBLs with AA (**e**) and AB (**f**) stacking. The scale bar is 3 μm. **g**, **h** ADF STEM images of the hBLs with AA (**g**) and AB (**h**) stacking. The insets are filtered images. The scale bar is 0.5 nm. **i**, **j** Schematics of the top and side views of the atomic registries of AA (**i**) and AB (**j**) stacking, according to the ADF STEM results. The AA stacking corresponds to the 3R-like stacking (**i**), with Se atoms of WSe_2_ on top of Mo atoms of MoSe_2_. The AB stacking corresponds to the 2H-like stacking (**j**), with W (Se) atoms of WSe_2_ on top of Se (Mo) atoms of MoSe_2_ layer

Our samples were grown on sapphire substrates (see Methods), containing WSe_2_/MoSe_2_ hBLs with parallel (AA) and antiparallel (AB) stacking, which have been identified by second harmonic generation (SHG) and annular dark-field (ADF) scanning transmission electron microscopy (STEM). In general, the hBLs consist of a monolayer WSe_2_ covering on top of a monolayer WSe_2_–MoSe_2_ lateral heterojunction^22, 23^ formed by a MoSe_2_ inner triangle with WSe_2_ epitaxially grown at the outer region. Figure 1a, b shows the optical images of hBL flakes with AA and AB stacking, respectively. PL, Raman, and atomic force microscopy characterizations (Supplementary Figs. 1, 2 and Supplementary Note 1) conclude that the inner triangles of both types are WSe_2_/MoSe_2_ hBLs, while the outer regions of AA (AB) stacking are monolayer (bilayer) WSe_2_, as schematically shown in Fig. 1c, d. We identified the stacking orientations (twist angle *θ*) by polarization-resolved SHG (Supplementary Fig. 3). In Fig. 1e, f, we show the SHG intensity maps of the AA- and AB-stacked hBLs, as shown in Fig. 1a, b, respectively. The strongly enhanced (suppressed) SHG intensity in the hBL regions is a clear evidence of AA (AB) stacking with *θ*=0° (*θ*=60°), due to the constructive (destructive) interference of SH fields from the individual layers^24^. We noted that the SHG intensity in the AB-stacked hBL region is not fully suppressed as that in the WSe_2_ bilayer region. The residual SHG intensity might be caused by the different SHG efficiencies of the two materials and the nonvanishing vertical dipole moments in the hBL regions excited and collected through the large-numerical-aperture objective. The atomic registries of the AA and AB stacking have been further identified by ADF STEM. As shown in Fig. 1g, h, the AA-stacked hBLs exhibit the 3R-like stacking (with Se atoms on top of Mo atoms), while the AB-stacked hBLs show the 2H-like stacking, as illustrated in Fig. 1i, j. Selected-area electron diffraction in the hBL region (~1-μm diameter) shows only one set of diffraction patterns (Supplementary Fig. 4 and Supplementary Note 2), which confirms that the WSe_2_/MoSe_2_ hBLs formed directly by CVD growth exhibit long-range stacking order without interlayer twists. The correlation between the morphology and stacking orientation thus facilitates the investigation of how the atomic registry affects the optical properties of WSe_2_/MoSe_2_ hBLs.

### Stacking-dependent interlayer excitons in WSe_2_/MoSe_2_ heterobilayers

**Fig. 2:**
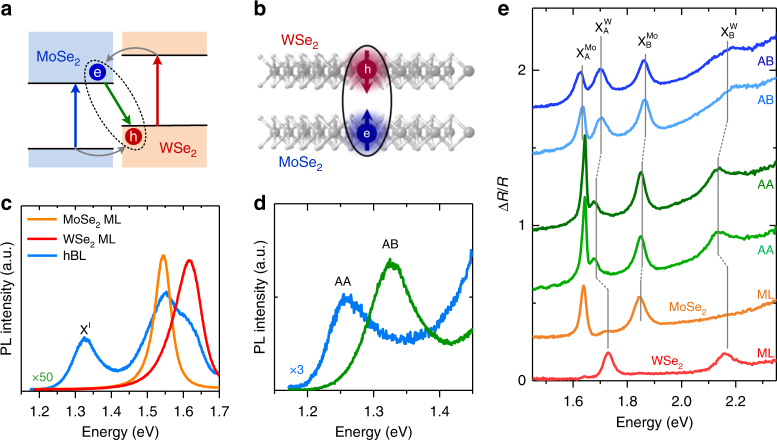
Interlayer excitons in WSe_2_/MoSe_2_ hBLs with AA and AB stacking. **a** The type-II band alignment of WSe_2_/MoSe_2_ hBLs. **b** Schematics of interlayer excitons with electrons (e) and holes (h) located in the MoSe_2_ and WSe_2_ layers, respectively. **c** PL spectra for monolayer WSe_2_, MoSe_2_, and the WSe_2_/MoSe_2_ hBL with AB stacking. The interlayer exciton X^I^ is observed at 1.33 eV. **d** A comparison of X^I^ peaks in hBLs with AA and AB stacking. The PL spectra shown in **c** and **d** were measured at room temperature (*T*=300 K). **e** Differential reflectance spectra Δ*R*/*R* for monolayer (ML) WSe_2_, ML MoSe_2_, and hBLs with AA and AB stacking. The Δ*R*/*R* spectra were measured at *T*=4 K. ${\mathrm{X}}_{\mathrm{A}}^{{\mathrm{Mo}}}$ and ${\mathrm{X}}_{\mathrm{A}}^{\mathrm{W}}$(${\mathrm{X}}_{\mathrm{B}}^{{\mathrm{Mo}}}$ and ${\mathrm{X}}_{\mathrm{B}}^{\mathrm{W}}$) denote A (B) excitons in MoSe_2_ and WSe_2_, respectively. For each stacking, two spectra from different hBL flakes are displayed in order to demonstrate the consistent spectral features

The WSe_2_/MoSe_2_ hBLs are known to exhibit a type-II band alignment^25, 26^ with the conduction band minimum (valence band maximum) located in the MoSe_2_ (WSe_2_) layer (Fig. 2a). Coulomb-bound electrons and holes localized in different monolayers thus form interlayer excitons (Fig. 2b). Figure 2c shows the room-temperature PL spectra for the monolayer WSe_2_, MoSe_2_, and the hBL with AB stacking. The PL emission at 1.62 eV (1.55 eV) corresponds to the excitonic states in monolayer WSe_2_ (MoSe_2_)^27–30^. In the hBL regions, the intralayer exciton peaks are also observed, but the intensities were quenched by a factor of ~50–100 due to the efficient interlayer carrier transfers^9–13^. In addition, we observed a lower energy peak at 1.33 eV, which is attributed to the interlayer exciton (X^I^) recombination^6, 7^. Specifically, we found that the X^I^ peak of AA-stacked hBLs is redshifted by ~70 meV in comparison with that of AB-stacked hBLs (Fig. 2d and Supplementary Fig. 5). Band structure calculations based on density functional theory (DFT) show that the MoSe_2_ bands in AA stacking exhibit a rigid downshift by ~60 meV in comparison with those in AB stacking (Supplementary Fig. 6 and Supplementary Note 3). The enlarged valence band offset and the spin splitting in the MoSe_2_ conduction band thus account for the redshift of the X^I^ peak in AA stacking^31^. Apart from X^I^, we found that the energies of intralayer excitons also change systematically with the atomic registry (Fig. 2e). The observed energy shifts of A and B excitons in monolayer WSe_2_ (${\mathrm{X}}_{\mathrm{A}}^{\mathrm{W}}$ and ${\mathrm{X}}_{\mathrm{B}}^{\mathrm{W}}$) and MoSe_2_ (${\mathrm{X}}_{\mathrm{A}}^{{\mathrm{Mo}}}$ and ${\mathrm{X}}_{\mathrm{B}}^{{\mathrm{Mo}}}$) agree qualitatively with the calculated band gap variation in hBLs with AA and AB stacking (Supplementary Fig. 7 and Supplementary Note 4). It has been established that the Moiré periodicity of incommensurate hBLs can induce band-gap modulations in the constituent monolayers^20, 21^. However, such periodicity cannot be resolved optically due to the limited spatial resolution. The agreement between the variations in the intralayer exciton energies and the calculated band gaps also suggests that the investigated WSe_2_/MoSe_2_ hBLs exhibit long-range stacking orders and well-defined atomic registries.

### Valley polarization of interlayer excitons

**Fig. 3:**
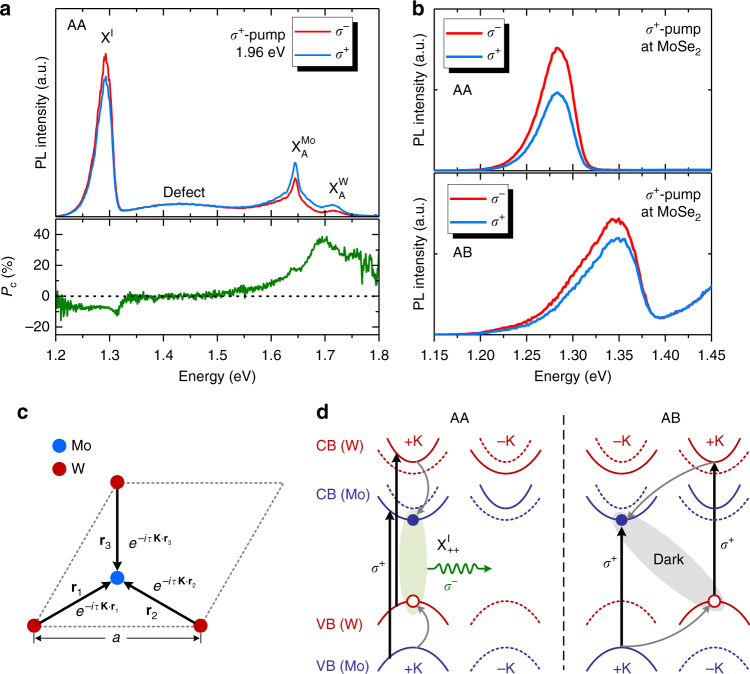
Valley polarization of interlayer excitons. **a** Top: polarization-resolved PL spectra for the AA-stacked hBL using *σ*^+^ excitation at 1.96 eV. Bottom: the degree of circular polarization *P*_C_. **b** Polarization-resolved PL spectra near the X^I^ peak using *σ*^+^ excitation at 1.64 eV (MoSe_2_) in AA- (top) and AB- (bottom) stacked hBLs. **c** The atomic registries and the phases associated with the interlayer transition dipoles in AA and AB stacking. A distinct phase factor $e^{i{\mathrm{\tau }}{\mathbf{K}} \cdot {\mathbf{r}}_n}$ is associated with each interlayer transition dipoles between the nearest Mo and W atoms in different layers. **d** Left: the formation of ${\mathrm{X}}_{ + + }^{\mathrm{I}}$ state in AA-stacked hBLs using *σ*^+^ excitations at +K valleys in MoSe_2_ and WSe_2_ layers. Right: the formation of the interlayer dark state in AB-stacked hBLs using *σ*^+^ excitations at both MoSe_2_ and WSe_2_ layers. Red and blue lines are WSe_2_ and MoSe_2_ bands, respectively. Solid and dotted lines represent bands with different spins. Vertical arrows indicate optical excitations. Gray arrows represent spin-conserving interlayer transfer to the lowest energy band

We examined the effect of atomic registry on the polarization properties of X^I^. Figure 3a shows the polarization-resolved PL spectra measured at *T*=4 K for the AA-stacked hBL using *σ*^+^ excitations at 1.96 eV (Supplementary Fig. 8 for AB hBLs). The intralayer exciton PL from the MoSe_2_ and WSe_2_ layers exhibits a stronger *σ*^+^ PL component, indicative of generating valley excitons at +K valleys in each layer by the above-gap *σ*^+^ excitations^1, 2^. These intralayer excitons then relax to form X^I^ through the electron and hole transfers across the WSe_2_/MoSe_2_ interface. If the spin and/or valley indices of carriers are preserved after interlayer transfer, valley-specific X^I^ can be formed. Interestingly, we found that the X^I^ PL also exhibits circular polarization, but with an opposite helicity, i.e., a stronger *σ*^−^ PL component under *σ*^+^ excitations. We define the degree of circular polarization as *P*_C_ = (*I*_+_ − *I*_−_)/(*I*_+_ + *I*_−_), where *I*_+_ (*I*_–_) denotes the intensity of co-polarized (cross-polarized) PL component with the excitation. As shown in Fig. 3a, the intralayer exciton peak in WSe_2_ (MoSe_2_) shows $P_{\mathrm{C}} \simeq 36\%$ ($P_{\mathrm{C}} \simeq 18\%$), while the X^I^ peak exhibits $P_{\mathrm{C}} \simeq - 7\%$. Using resonant excitation with the A exciton energy of MoSe_2_ (1.64 eV) markedly increases the *P*_c_ of X^I^ up to $\simeq - 23\%$ (Fig. 3b). Using *σ*^+^ excitations at the MoSe_2_ layer in AB-stacked hBLs, the X^I^ PL also exhibits a stronger *σ*^−^ PL component, but with a smaller $P_{\mathrm{C}} \simeq - 9\%$.

### Interlayer quantum interference

The negative circular polarization arises from the interlayer quantum interference imposed by the atomic registry between the WSe_2_ and MoSe_2_ layers. We analyze the polarization properties of X^I^ based on the theory proposed by Yu et al.^17^. The valley configurations of X^I^ can be classified as ${\mathrm{X}}_{\tau \prime \tau }^{\mathrm{I}}$, i.e., electron (hole) at *τ*′K (*τ*K) valley in the MoSe_2_ (WSe_2_) layer, where *τ*^'^,*τ* = ±1 are the valley index. The Bloch function of the conduction (valence) band edge at *τ*'K (*τ*K) valley consists predominantly of the $d_{{\mathrm{z}}^2}$ ($d_{{\mathrm{x}}^2 - {\mathrm{y}}^2} + i\tau d_{{\mathrm{xy}}}$) orbitals with a magnetic quantum number *m* = 0 (*m* = 2*τ*) on Mo (W) sites in the MoSe_2_ (WSe_2_) layer. The interlayer transition dipole of ${\mathrm{X}}_{\tau \prime \tau }^{\mathrm{I}}$ can be expressed as ${\mathbf{D}}_{\tau \prime \tau } \cong \langle\psi _{{\mathrm{v}},\tau {\mathbf{K}}}^{\mathrm{W}}\left( {\mathbf{r}} \right){\mathrm{|}}{\hat{\mathbf D}}{\mathrm{|}}\psi _{{\mathrm{c}},\tau \prime {\mathbf{K}}}^{{\mathrm{Mo}}}\left( {\mathbf{r}} \right)\rangle$, which connects the orbitals on Mo and W sites in different layers^17^. As a first approximation, we consider the nearest-neighbor interlayer transition dipoles between Mo and W orbitals as shown in Fig. 3c. The total transition dipole is the superposition of the three dipoles associated with a distinct phase factor $e^{ - i\tau {\mathbf{K}} \cdot {\mathbf{r}}_n}$, i.e., ${\mathbf{D}}_{\tau \prime \tau } \propto \mathop {\sum }\limits_{n = 1,2,3} e^{ - i\tau {\mathbf{K}} \cdot {\mathbf{r}}_n}\left\langle {d_{m = 2\tau }^{\mathrm{W}}({\mathbf{r}}_n){\mathrm{|}}{\hat{\mathbf D}}{\mathrm{|}}d_{m = 0}^{{\mathrm{Mo}}}(0)} \right\rangle$, where ***r***_*n*_ is the vector pointing from W to Mo sites (Supplementary Note 5). The quantum interference imposed by the atomic registry thus gives rise to a distinct polarization selection rule for X^I^. In general, the interlayer transition dipole also acquires contributions from coupling to intralayer excitons via interlayer hopping^17^. Symmetry analysis indicates that the *σ*^+^ and *σ*^−^ components of ${\mathbf{D}}_{\tau \prime \tau }$ are^17^

1 $$\begin{array}{l}{\mathbf{e}}_\tau \cdot {\mathbf{D}}_{\tau \prime \tau } \propto e^{ - i\tau {\mathbf{K}} \cdot {\mathbf{r}}_1} + e^{ - i\tau {\mathbf{K}} \cdot {\mathbf{r}}_2} + e^{ - i\tau {\mathbf{K}} \cdot {\mathbf{r}}_3},\\ {\mathbf{e}}_{ - \tau } \cdot {\mathbf{D}}_{\tau \prime \tau } \propto e^{ - i\tau {\mathbf{K}} \cdot {\mathbf{r}}_1} + e^{ - i\tau({\mathbf{K}} \cdot {\mathbf{r}}_2 + 2\pi /3)} + e^{ - i\tau({\mathbf{K}} \cdot {\mathbf{r}}_3 + 4\pi /3)}\end{array}$$

where **e**_±_ = (*x*±*iy*)/(2)^1/2^ is the unit vector of *σ*^±^ polarization. From the valley optical selection rule, the PL helicity for the four possible valley configurations of bright X^I^ states in hBLs with AA and AB stacking can be determined (Supplementary Fig. 9). Since *σ*^+^ excitation creates intralayer excitons at +K valleys in both MoSe_2_ and WSe_2_, a majority of ${\mathrm{X}}_{ + + }^{\mathrm{I}}$ is expected to form in AA-stacked hBLs via spin-conserving interlayer hopping (Fig. 3d). For ${\mathrm{X}}_{ + + }^{\mathrm{I}}$ in AA stacking, the quantum interference cancels out the *σ*^+^ component (**e**_+_ ⋅ **D**_+ +_ = 0) but with the nonvanishing *σ*^−^ component (**e**_−_ ⋅ **D**_+ +_ ≠ 0), giving rise to a net *σ*^−^-polarized PL. The low PL polarization ($P_{\mathrm{C}} \simeq - 7\%$) of X^I^ created by excitation at 1.96 eV arises from the valley depolarization of intralayer excitons before the formation of X^I^. Resonant excitation with the exciton energy in MoSe_2_ (1.64 eV) considerably reduces the intralayer valley depolarization and hence increases the X^I^ PL circular polarization.

### Excitation energy dependence of emission polarization

**Fig. 4:**
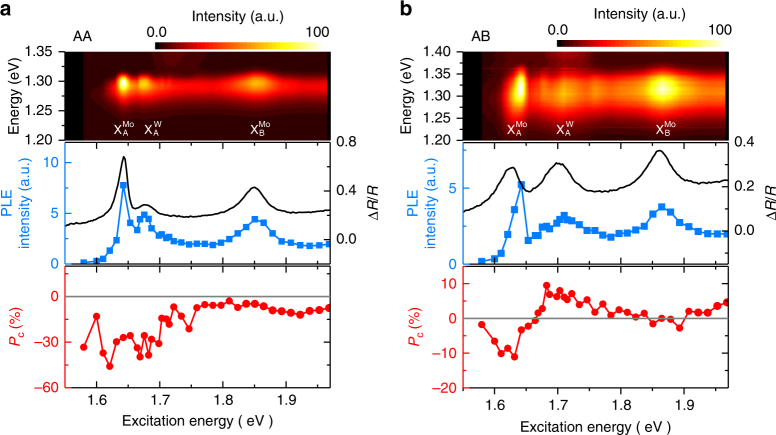
Excitation energy dependence of PL intensity and circular polarization of interlayer excitons. **a** AA stacking. **b** AB stacking. Top: contour plots for the X^I^ PL spectra using different excitation energies. Middle: the corresponding PLE spectra. The differential reflectance spectra Δ*R*/*R* are also shown for comparison. Bottom: the degree of circular polarization *P*_C_ as a function of excitation energy. ${\mathrm{X}}_{\mathrm{A}}^{{\mathrm{Mo}}}$ and ${\mathrm{X}}_{\mathrm{A}}^{\mathrm{W}}$(${\mathrm{X}}_{\mathrm{B}}^{{\mathrm{Mo}}}$ and ${\mathrm{X}}_{\mathrm{B}}^{\mathrm{W}}$) denote A (B) excitons in MoSe_2_ and WSe_2_, respectively. The ${\mathrm{X}}_{\mathrm{A}}^{\mathrm{W}}$ peak measured by PLE and differential reflectance Δ*R*/*R *is dominated by trion absorption

We performed PL excitation (PLE) spectroscopy to examine the energy relaxation channels for the formation of X^I^. Enhanced X^I^ PL emission was observed when the excitation energy (*E*_ex_) is resonant with intralayer excitons (Fig. 4a, b), indicating that X^I^ is formed via energy relaxation from intralayer excitons created in either MoSe_2_ or WSe_2_ layers. In AA stacking, pronounced negative *P*_C_ for X^I^ is observed when *E*_ex_ < 1.73 eV, regardless of creating valley excitons in either layer. Since the K valleys of both layers are aligned in momentum space for AA stacking, the direct interlayer spin–valley transfer ensures that a majority of ${\mathrm{X}}_{ + + }^{\mathrm{I}}$ is formed via *σ*^+^ excitation at either the MoSe_2_ or the WSe_2_ layer. In AB stacking, however, the ±K valleys of MoSe_2_ are aligned with the opposite ∓K valleys of WSe_2_ in momentum space. The preferential spin–valley configurations of X^I^ in AB stacking are therefore different. As shown in Fig. 4b, the X^I^ PL also shows *P*_C_ < 0 when creating valley excitons in the MoSe_2_ layer (*E*_ex_ <1.65 eV), but becomes *P*_c_ > 0 when switching the excitation to the WSe_2_ layer (*E*_ex_~ 1.7 eV).

## Discussion

According to the valley optical selection rule for AB stacking, the negative (positive) *P*_c_ indicates the emission of *σ*^−^ (*σ*^+^) PL from the bright ${\mathrm{X}}_{ - + }^{\mathrm{I}}$ (${\mathrm{X}}_{ + - }^{\mathrm{I}}$) state formed by *σ*^+^ excitation at the MoSe_2_ (WSe_2_) layer. It has been established experimentally that the interlayer charge-transfer process is dominated by a spin-conserving transfer to the lowest energy band, independent of the interlayer momentum mismatch^32^. Our measurements based on nondegenerate optical circular dichroism (CD) spectroscopy on the AA- and AB-stacked hBLs also support this picture (Supplementary Note 6 and Supplementary Figs. 10–13). For *σ*^+^ pump at the MoSe_2_ layer with AB stacking, the spin-conserving transfer thus leads to the lowest valley configuration ${\mathrm{X}}_{ + + }^{\mathrm{I}}$ (Fig. 3d) in the steady state. The ${\mathrm{X}}_{ + + }^{\mathrm{I}}$ state is an intervalley dark state (Supplementary Table 1) with a large center-of-mass momentum (±ℏ**K**), which is far beyond the light cone and unable to couple with light directly. The formation of bright X^I^ states (${\mathrm{X}}_{ - + }^{\mathrm{I}}$ or ${\mathrm{X}}_{ + - }^{\mathrm{I}}$) is expected to occur by intervalley or intravalley scattering processes (Supplementary Note 7 and Supplementary Fig. 14a,b). Since the electron intervalley scattering without spin flips is expected to be more efficient, a majority of the ${\mathrm{X}}_{ - + }^{\mathrm{I}}$ will form under *σ*^+^ excitation at the MoSe_2_ layer, giving rise to the *σ*^−^ PL emissions (Supplementary Fig. 14a). However, the presence of the lower-lying dark state makes the bright X^I^ states in AB stacking energetically unfavorable, rendering less-efficient PL at low temperatures (Supplementary Fig. 15). As for *σ*^+^ pump at the WSe_2_ layer with AB stacking, the spin-conserving interlayer transfer also leads to a majority of dark ${\mathrm{X}}_{ + + }^{\mathrm{I}}$ in the steady state. However, the microscopic processes for the formation of bright X^I^ states are intrinsically more complicated because resonant excitations at the WSe_2_ layer also inject carriers nonresonantly to the MoSe_2_ layer. The formation of bright ${\mathrm{X}}_{ + - }^{\mathrm{I}}$ state thus requires spin-flip processes (Supplementary Fig. 14c), which is expected to be energetically unfavorable. Nevertheless, the low circular polarization for X^I^ in AB-stacked hBL suggests that there is a competing channel for the formation of ${\mathrm{X}}_{ - + }^{\mathrm{I}}$ states. While the microscopic processes remain unclear, a possible scenario is likely mediating through the generation of intralayer trions in the WSe_2_ layer. This explanation is also supported by the PLE spectra for AB-stacked hBLs (Fig. 4b), where the excitation resonances of higher *P*_C_ are lower than the neutral exciton energy in monolayer MoSe_2_ and WSe_2_. Further studies, such as using time-resolved Kerr rotation spectroscopy^33^, are required in order to understand the microscopic processes for the formation dynamics of bright X^I^ states.

In summary, we unveil the polarization properties of interlayer excitons in commensurate WSe_2_/MoSe_2_ heterobilayers with a well-defined atomic registry. The quantum interference imposed by interlayer atomic registry gives rise to distinct polarization selection rules for interlayer excitons, making the interlayer valley configurations become optically traceable. Selective excitations at different monolayers further demonstrate the optical addressability of interlayer excitons with different valley configurations and polarization helicities, providing a new scheme for exploring spin/valleytronic applications based on van der Waals heterostructures.

## Methods

### Material synthesis of WSe_2_/MoSe_2_ heterobilayers

High-quality single-crystal WSe_2_/MoSe_2_ heterobilayers were synthesized on sapphire substrates by chemical vapor deposition (CVD) in a horizontal hot-wall chamber using the conventional one-pot synthesis process^34, 35^. High-purity MoO_2_ (99%, Aldrich), WO_3_, and Se powders (99.5%, Alfa) were used as the initial reactants. The heterostructures were grown at 880 °C in Ar/H_2_ flowing gas at low pressure (5–40 Torr). The flow rates for Ar/H_2_ gas were controlled at 60/6 sccm during the growth.

### ADF STEM characterizations

ADF STEM imaging was conducted using a spherical aberration-corrected transmission electron microscope (JEOL-2100F). The CVD-grown WSe_2_/MoSe_2_ flakes on the substrate were first capped with a layer of poly(methylmethacrylate) (PMMA) (950K A4) by spin-coating (step 1: 500 rpm for 10 s; step 2: 3000 rpm for 60 s), followed by baking at 100 °C for 60 min. The PMMA-capped WSe_2_/MoSe_2_ was then immersed in a BOE solution at 100 °C for 60 min. After that, the PMMA film can be exfoliated from the sapphire substrate and transferred onto a Cu grid with carbon nets (Ted Pella) after diluting etchants and residues in deionized water. Then the top PMMA film was removed by acetone, and the sample was cleaned by isopropyl alcohol and deionized water.

### Optical measurements

Room-temperature optical characterizations, such as photoluminescence (PL), Raman, SHG, and differential reflectance spectroscopes were performed using a homebuilt optical microscope in the back-scattering configuration. The excitation light was focused onto the sample by a ×100 objective lens (N.A. = 0.9). The signals were collected by the same objective lens and analyzed by a 0.75-m monochromator and detected by a liquid-nitrogen-cooled CCD camera. For PL and Raman measurements, a 532-nm solid-state laser was used as the excitation source. For differential reflectance measurements, a fiber-coupled tungsten–halogen lamp was used as a white-light source. For SHG measurements, the fundamental laser field was provided by a mode-locked Ti:sapphire laser at 880 nm. Spatial mappings were performed on a fast motorized *x*–*y* stage with a step of 0.25 μm. The polarizations of fundamental and SH lights were selected and analyzed by individual linear polarizers and half-wave plates.

For low-temperature PL measurements, the sample was cooled down to *T* = 4 K by a cryogen-free low-vibration cryostat equipped with a three-axis piezo-positioner, an *x*–*y* scanner, and an objective lens (N.A. = 0.82) in the low-temperature chamber. Excitations at three different energies, corresponding to 1.96 (HeNe laser), 1.71, and 1.64 eV (cw-tunable Ti:sapphire laser), were used for above-gap and resonant excitations in the WSe_2_ and MoSe_2_ layers. For PL excitation (PLE) measurements, we used a supercontinuum laser equipped with a continuous tunable filter as the excitation source. The bandwidth of the tunable filter is ~1–2 nm. For polarization-resolved PL measurements, the circular polarization of the excitation laser was selected by a set of linear polarizers and quarter-wave plates, and the PL polarizations were analyzed by another set of quarter-wave plates and linear polarizers in front of the grating spectrometer. We have calibrated the circular polarization in both the detection and excitation paths. In the detection path, the degree of circular polarization is preserved up to 99.1% after passing through all optical components in the optical path. In the excitation path, the circular polarization of the excitation laser is higher than 98%.

### Band structure calculations

The ab initio calculations were carried out using the Quantum ESPRESSO software^36^ with the LDA pseudopotentials^37^. An 18 × 18 × 1 *k*-grid is used to sample the Brillouin zone, and the energy cutoff of the plane-wave expansion is 50 Ry. Crystal structures were fully relaxed with the inclusion of the van der Waals correction to the interatomic forces by means of the DFT-D2 method^38^. Spin–orbit coupling was included in all calculations.

### Data availability

Data described in this paper and presented in the supplementary materials are available from the corresponding author upon request.

## Electronic supplementary material

Supplementary Information
