# Supplementary material for: Negative circular polarization emissions from WSe2/MoSe2 commensurate heterobilayers
Source: Nat Commun. 2018 Apr 10;9:1356. doi: 10.1038/s41467-018-03869-7 (PMC5893569; doi:10.1038/s41467-018-03869-7)
Supplement: Supplementary file 1 — Supplementary Information [file 41467_2018_3869_MOESM1_ESM.pdf]

Supplementary Information for

Negative circular polarization emissions from $WSe_2/MoSe_2$ commensurate heterobilayers

Wei-Ting Hsu[1], Li-Syuan Lu[1], Po-Hsun Wu[1], Ming-Hao Lee[2], Peng-Jen Chen[3], Pei-Ying Wu[1], Yi-Chia Chou[1], Horng-Tay Jeng[4], Lain-Jong Li[5], Ming-Wen Chu[2], and Wen-Hao Chang[1,6]*

[1]Department of Electrophysics, National Chiao Tung University, Hsinchu 30010, Taiwan.

[2]Center for Condensed Matter Sciences, National Taiwan University, Taipei 10617, Taiwan.

[3]Institute of Physics, Academia Sinica, Taipei 11529, Taiwan.

[4]Department of Physics, National Tsing Hua University, Hsinchu 30010, Taiwan.

[5]Physical Sciences and Engineering, King Abdullah University of Science and Technology, Thuwal, 23955-6900, Kingdom of Saudi Arabia.

[6]Center for Emergent Functional Matter Science, National Chiao Tung University, Hsinchu 30010, Taiwan

*Corresponding author: E-mail: whchang@mail.nctu.edu.tw

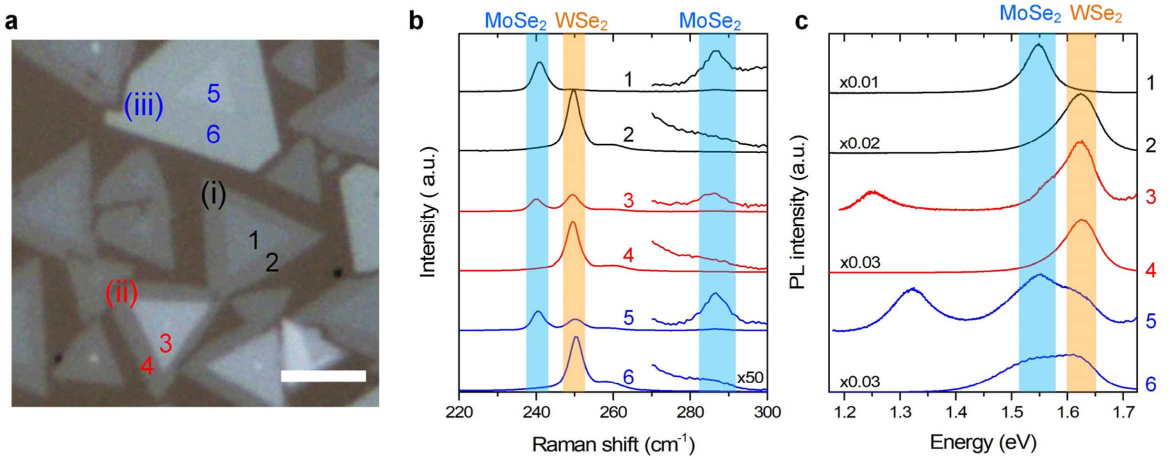

Supplementary Figure 1: Identification of stacking structures by Raman and PL spectroscopies. a, Optical image of three different heterostructure flakes on the same substrate. The scale bar is 5 μm. **b,c,** Raman (**b**) and PL (**c**) spectra measured at different regions labeled in (**a**). Regions 3 and 5 are identified as $WSe_2/MoSe_2$ hBLs.

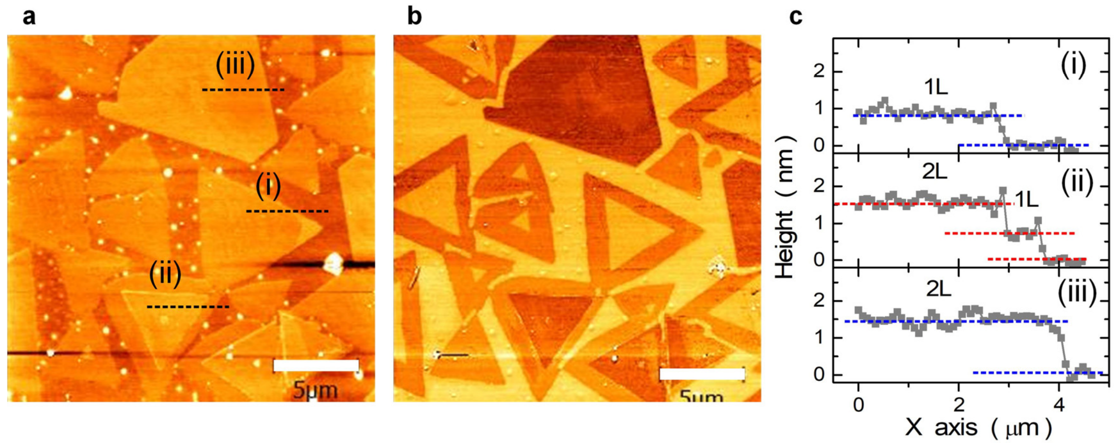

Supplementary Figure 2: Determination of layer number by AFM. a,b, AFM morphology (**a**) and phase (**b**) maps for $WSe_2/MoSe_2$ heterostructures. The scale bar is 5 μm. **c,** The height profiles of line scans (i) to (iii) indicated in (a).

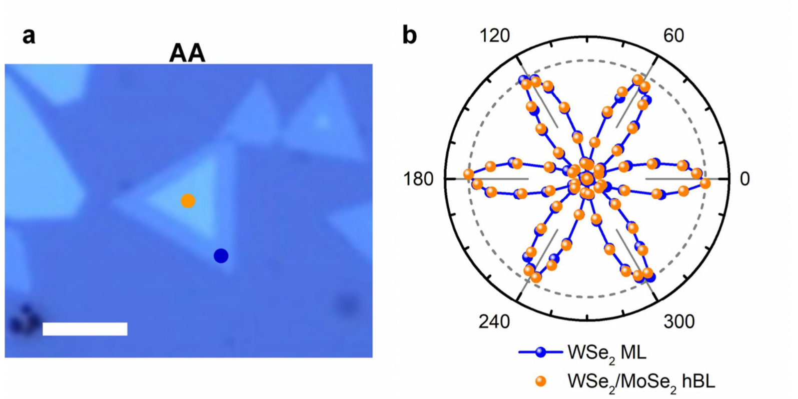

Supplementary Figure 3: Polarization-resolved SHG. a, The optical microscope image of an AA-stacked hBL flake. The scale bar is 5 μm. **b,** The polar plot of the normalized intensity of polarization-resolved SHG from the WSe_2 monolayer (blue dots) and the $WSe_2/MoSe_2$ hBL region (orange dots) as a function of azimuthal angle φ.

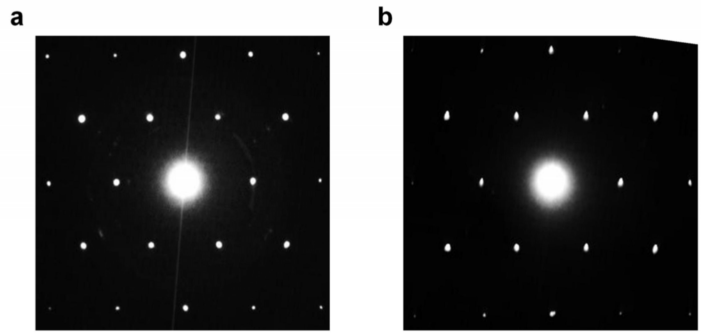

Supplementary Figure 4: SAED patterns of hBL regions. a, AA stacking. **b,** AB stacking.

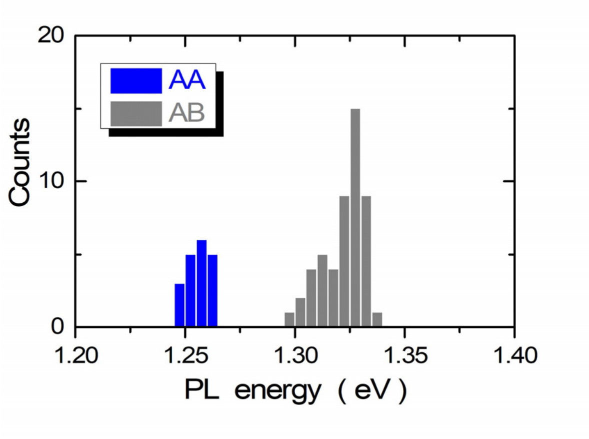

Supplementary Figure 5: Histogram of X^I PL peak energy in hBLs with AA and AB stacking. The histogram collects data from 19 flakes with AA stacking (blue bars) and 50 flakes with AB stacking (gray bars) on the same substrate. The measurements were conducted at room temperature (300 K).

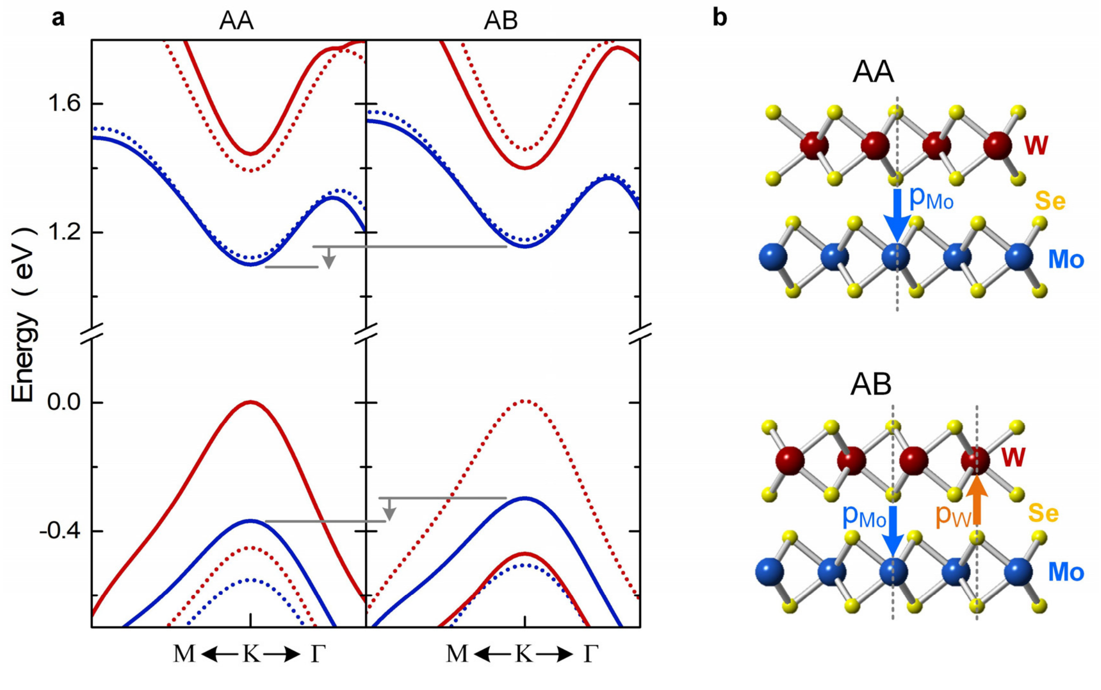

Supplementary Figure 6: a, Calculated band structures of WSe_2/$MoSe_2$ hBLs with AA (left) and AB (right) stacking. Red and blue lines are WSe_2 and $MoSe_2$ bands, respectively. Solid and dotted lines represent bands with different spins. **b,** Interlayer atomic registries of AA and AB stacking with 3R-like and 2H-like stacking, respectively. The AA stacking exhibit a net dipole pointing from the WSe_2 to the $MoSe_2$ layer.

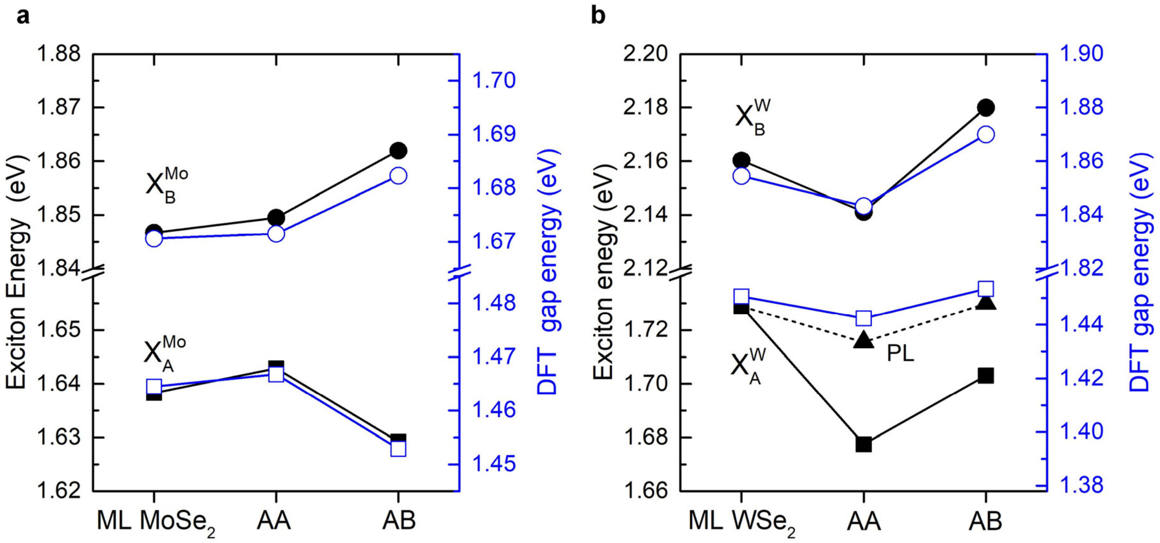

Supplementary Figure 7: Comparisons of the measured intralayer exciton energy determined by differential reflectance spectra and the calculated bandgap energy in monolayer $MoSe_2$, WSe_2 and hBLs with AA and AB stacking. **a,** Excitons (solid symbols) and bandgap (open symbols) of $MoSe_2$. **b,** Excitons (solid symbols) and bandgap (open symbols) of WSe_2. Neutral exciton energy of WSe_2 determined by PL are also included (solid triangles).

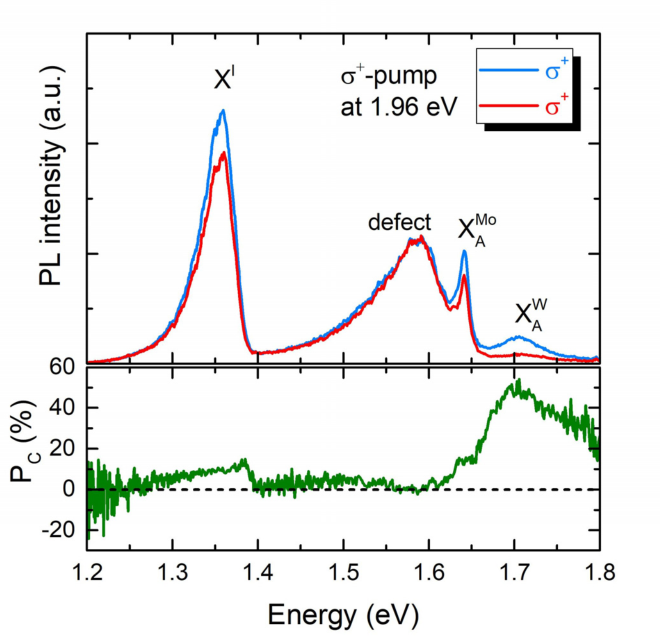

Supplementary Figure 8: Polarization-resolved PL for the AB stacked hBL. Top: Circular polarization-resolved PL spectra using σ^+ excitation at 1.96 eV. Bottom: Positive circular polarizations for both of the intralayer and interlayer excitons are observed. The broad PL emission band between X_A^{Mo} and X^I is attributed to the emissions from defect-bound excitons in monolayer WSe_2, since this PL band shows no valley polarization and exhibits the largest intensity when excitation at WSe_2 exciton energy. The X^I PL shows positive P_C, in contrast to the negative P_C shown in **Fig. 3b** using σ^+ excitation at 1.64 eV ($MoSe_2$ exciton resonance).

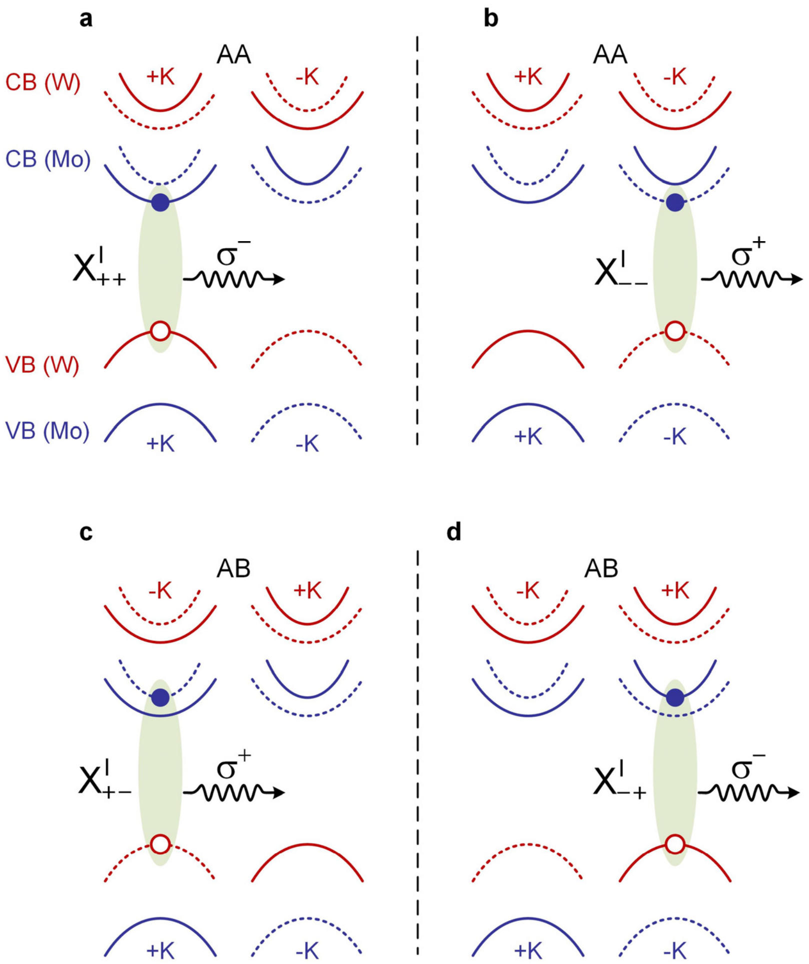

Supplementary Figure 9: Polarization selection rule for bright X^I states. **a,b,** AA stacking. **c,d,** AB stacking.

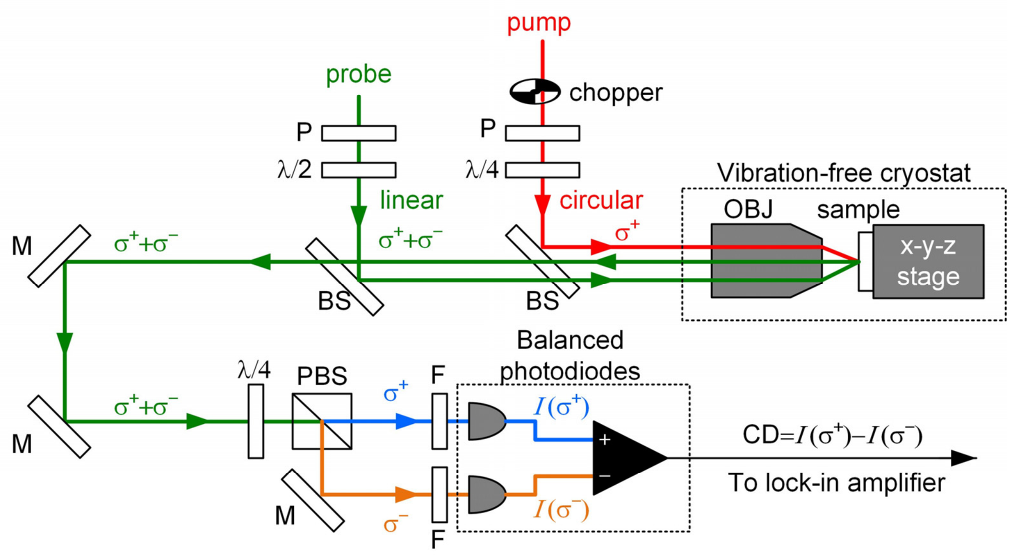

Supplementary Figure 10: Experimental setup for CD measurements. A circularly-polarized pump beam from a cw Ti:Sapphire laser was used to create spin-valley polarized carriers in the $MoSe_2$ and the WSe_2 layers. A linearly-polarized probe beam from a wavelength-tunable supercontinuum laser was used as the probe for the pump induced CD spectra. Both the pump and probe were focused to the sample by an objective lens (OBJ). The σ^+ and σ^- components of the reflected probe beam were separated by a quarter-wave plate (λ/4) in conjunction with a polarization beam splitter (PBS) and detected by a pair of balanced photodiodes. The pump beam signals were rejected by short-pass or long-pass filters (F). The pump beam was also modulated by an optical chopper in order to enable lock-in detection for the CD spectra. P: polarizer; λ/2: half-wave plate; BS: beam splitter; M: mirror.

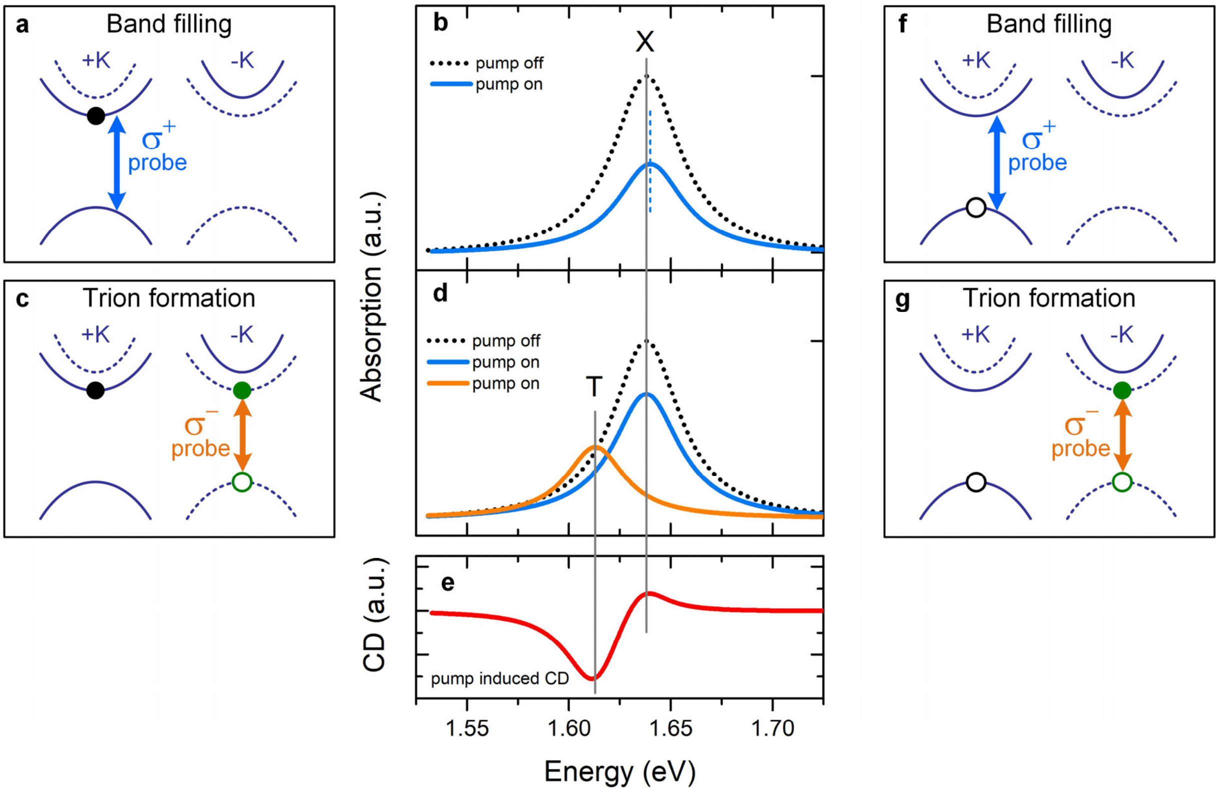

Supplementary Figure 11: CD response. a,b, Co-polarized σ^+ pump and σ^+ probe leads to the band filling effect. **c,d,** Cross-polarized σ^+ pump and σ^- probe leads to the trion formation. **e,** The CD spectra resulting from the difference between the co-polarized σ^+ probe and the cross-polarized σ^- probe. **g,h,** The effects of band filling (g) and trion formation (h) for valley-polarized holes in WSe_2.

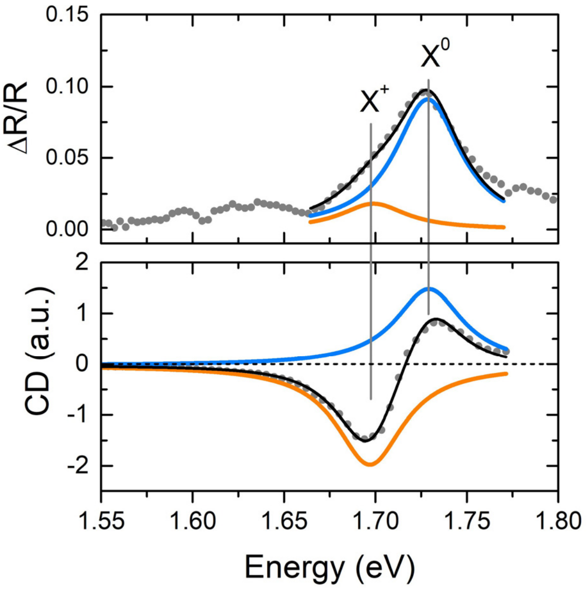

Supplementary Figure 12: CD spectrum of monolayer WSe_2. Bottom: The CD spectrum (gray dots) measured at the monolayer WSe_2 region using σ^+ pumping at 1.797 eV (690 nm). Top: the differential reflectance spectrum measured at the monolayer WSe_2 region. Black solid lines are fitted curves using two Lorentzian functions (blue and orange lines), corresponding to the exciton and trion absorptions.

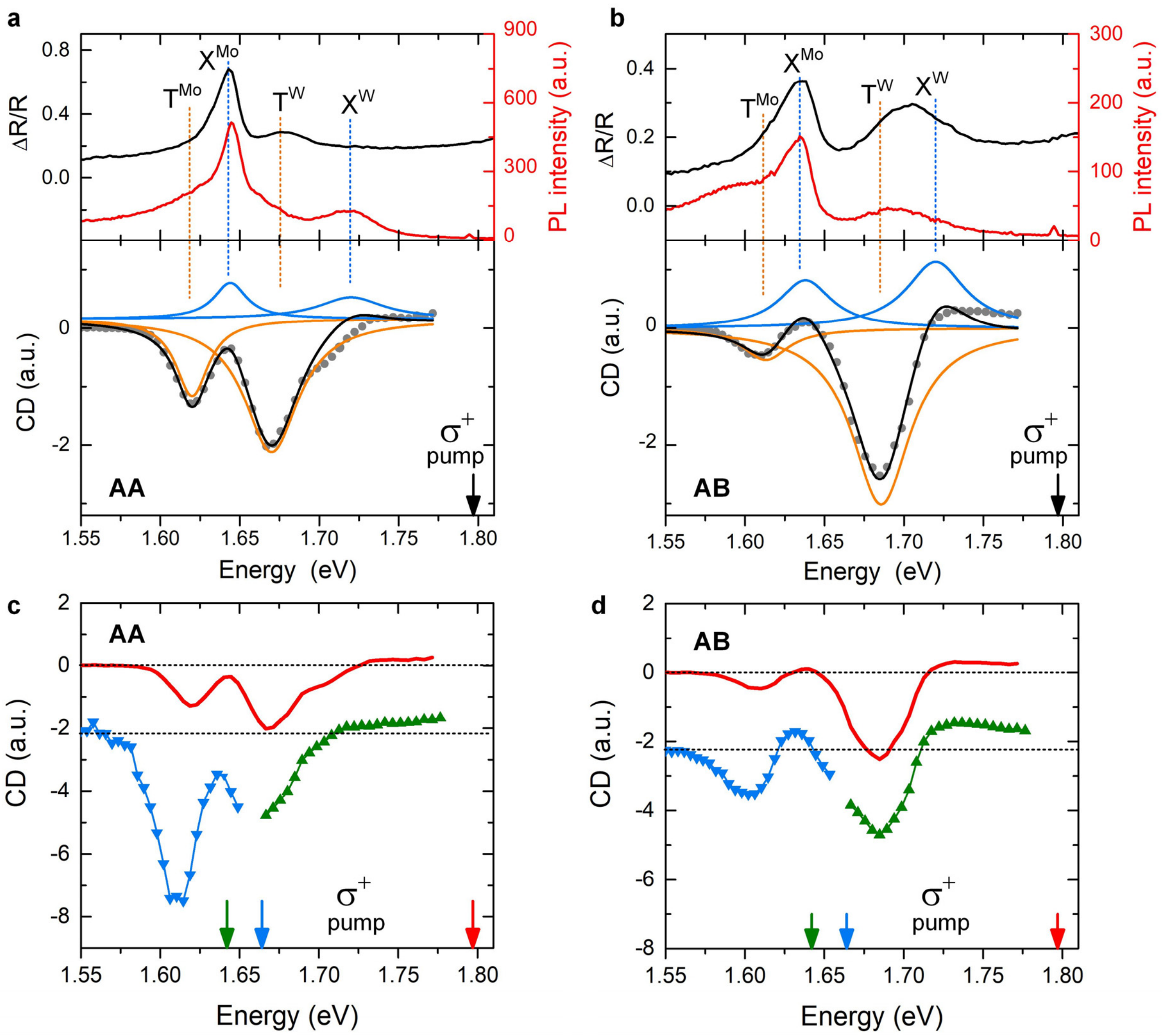

Supplementary Figure 13: CD spectra of AA and AB stacked hBLs. (**a,b**) Lower panel: Analysis of CD spectra (gray dots) measured from AA-stacked (**a**) and AB-stacked (**b**) hBLs using σ^+ pumping at 1.797 eV (690 nm). Black solid lines are fitted curves using two positive (blue) and two negative (orange) Lorentzian functions. Upper panel: the differential reflectance (black) and PL spectra (red). The positive (negative) peaks corresponds well with the exciton (trion) peaks X^W (T^W) and X^{Mo} (T^{Mo}) of WSe_2 and $MoSe_2$. (**c,d**). The CD spectra for AA and AB stacking measured using σ^+ pumping at different energies: 1.797 eV (red), 1.664 eV (blue) and 1.642 eV (green).

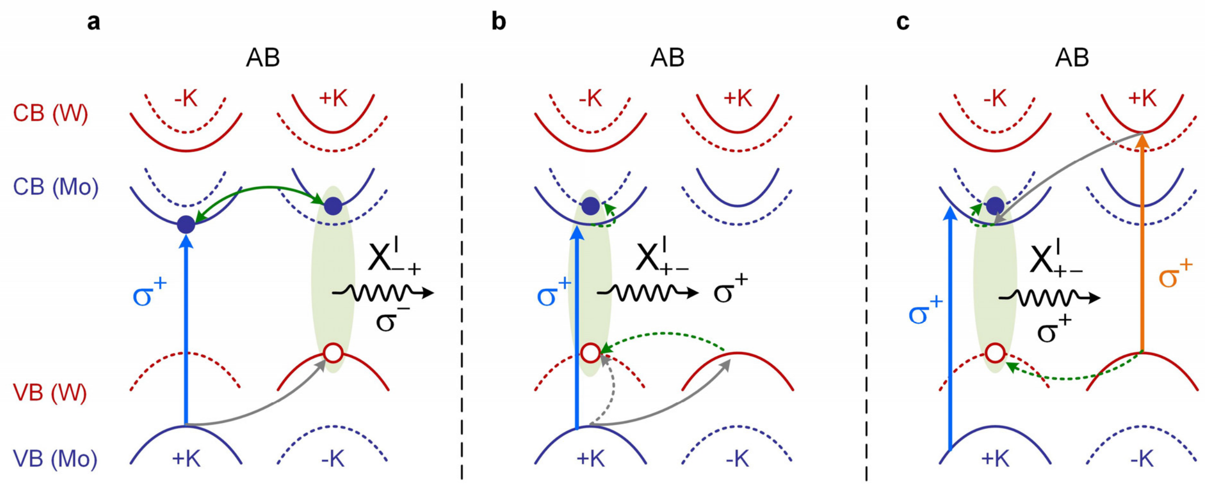

Supplementary Figure 14: The formation of bright X^I states in AB stacked hBLs. a,b, The formation of bright (a) X^I_{-+} and (b) X^I_{+-} states using σ^+ excitation at the $MoSe_2$ layer. Red and blue lines are WSe_2 and $MoSe_2$ bands, respectively. Solid and dotted lines represent bands with different spins. Vertical arrows indicate optical excitations. Grey solid (dotted) arrows represent interlayer transfer to the lowest energy band without (with) spin flips. Green solid (dotted) arrows represent intralayer scatterings without (with) spin flips. **c,** The formation of bright X^I_{+-} state using σ^+ excitation at the WSe_2 layer. Since the optical gap of WSe_2 is higher than that of $MoSe_2$, the σ^+ excitation also injects +K polarized carriers into $MoSe_2$ non-resonantly. The formation of X^I_{+-} state involves spin-flip processes.

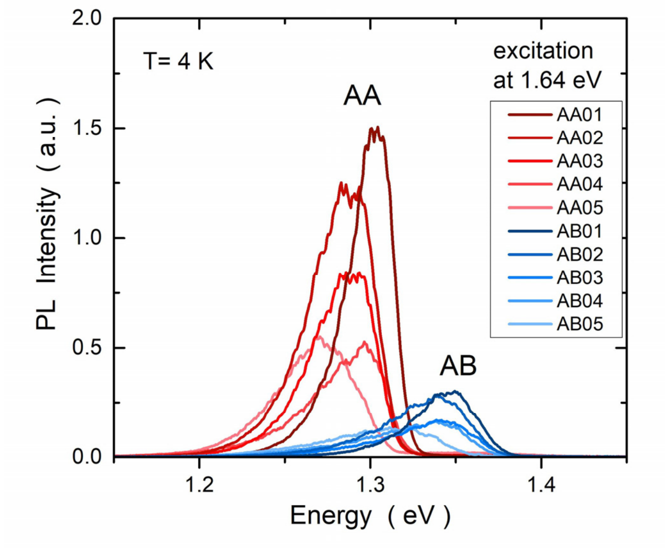

Supplementary Figure 15: PL spectra from ten hBLs with AA and AB stacking measured at 4 K. The PL intensity of X^I in AB stacking is generally weaker by a factor of 2-5 in comparison with that in AA stacking.

Supplementary Table 1: The helicity of circular polarization for the four possible valley configurations of bright X^{I} states in hBL with AA and AB stacking.

	X^{I}_{++}	X^{I}_{--}	X^{I}_{-+}	X^{I}_{+-}
AA	σ^{-}	σ^{+}	dark	dark
AB	dark	dark	σ^{-}	σ^{+}

Supplementary Note 1. Characterization of heterobilayers

Different types of heterostructure flakes were grown on the same substrate and have been characterized by PL and Raman spectroscopes. In **Supplementary Figure 1a**, we show a representative optical image containing three types of heterostructures. According to the optical contrast, we label regions 1-6 in the flakes (i) to (iii). The corresponding Raman and PL spectra are shown in **Supplementary Figure 1b,c**. From these spectra, we identified that regions 1 and 2 in flake (i) are monolayer $MoSe_2$ and WSe_2, respectively; regions 3 and 4 in flake (ii) are $WSe_2/MoSe_2$ hBL and monolayer WSe_2, respectively; regions 5 and 6 in flake (iii) are $WSe_2/MoSe_2$ hBL and bilayer WSe_2, respectively.

The layer numbers of these heterostructure flakes have also been characterized by atomic force microscopy (AFM), as shown in **Supplementary Figure 2**. From the line scan of height profiles across different regions in the flakes (i) to (iii), we confirm that flake (i) is monolayer lateral heterostructure; flake (ii) consists of an inner $WSe_2/MoSe_2$ hBL and an outer monolayer WSe_2; flake (iii) consists of an inner $WSe_2/MoSe_2$ hBL and an outer bilayer WSe_2.

Supplementary Note 2. Selected area electron diffraction

The selected area electron diffraction (SAED) patterns of the hBL regions were taken in a JEOL 2100F electron microscope operated at 200 kV. The heterostructure flakes were transferred onto a Cu grid with carbon nets using previously described transfer process. A selected-area aperture with an effective diameter of about 1 μm at the specimen was used. In **Supplementary Figure 4**, we show the SAED patterns of the AA (**Supplementary Figure 4a**) and AB (**Supplementary Figure 4b**) stacked hBLs, where only one set of diffraction patterns is observed, confirming that the $WSe_2/MoSe_2$ hBLs formed directly by CVD growth are coherently stacked without interlayer twists.

Supplementary Note 3. Calculated band structures for $WSe_2/MoSe_2$ hBL with different stacking

To understand how the interlayer lattice registrations affect the X^I energy, we performed band structure calculations for $WSe_2/MoSe_2$ hBLs with AA and AB stacking based on DFT. The atomic

registries of AA and AB stacking are based on the 3R-like and 2H-like stacking shown in **Fig. 1i,j**, respectively. In **Supplementary Figure 6a**, we show the calculated band structures near the K valley. The interlayer electronic couplings are generally weak near the K valleys due to the large valance band offset (Δ_{VBO}) between $MoSe_2$ and WSe_2 ($\Delta_{\mathrm{VBO}} \sim 0.3$ eV in AB stacking). For the AA-stacked hBL, the $MoSe_2$ bands exhibit a rigid down shift by ~60 meV in comparison with those in the AB-stacked hBL. This can be explained by the different interlayer atomic registries in AA and AB stacking. In AA stacking, the aligned Mo and Se atoms in different layers exhibits a net vertical dipole moment (**Supplementary Figure 6b**), giving rise to an electric field pointing from the $MoSe_2$ to the WSe_2 layers and hence enlarging the band offset. On the other hand, due to the coexistence of opposite Se-Mo and Se-W dipoles in AB stacking (**Supplementary Figure 6b**), the net vertical dipole moment is expected to be smaller. The enlarged Δ_{VBO} and the spin splitting in the $MoSe_2$ conduction band thus account for the redshift of $\mathrm{X^I}$ peak in AA stacking.

Supplementary Note 4. Intralayer exciton energy and calculated bandgap energy in $MoSe_2$ and WSe_2

From the differential reflectance spectra shown in **Fig. 2e**, the energy for intralayer excitons can be determined. Here we denote the A (B) exciton peak in $MoSe_2$ and WSe_2 as $\mathrm{X_A^{Mo}}$ and $\mathrm{X_A^{W}}$ ($\mathrm{X_B^{Mo}}$ and $\mathrm{X_B^{W}}$), respectively. In **Supplementary Figure 7**, we compare the measured intralayer exciton energy with the calculated bandgap energy in monolayer $MoSe_2$, WSe_2 and hBLs with AA (3R-like) and AB (2H-like) stacking. We found that the observed energy shifts of A- and B-exciton peaks in $MoSe_2$ ($\mathrm{X_A^{Mo}}$ and $\mathrm{X_B^{Mo}}$) and the B-exciton peak in WSe_2 ($\mathrm{X_B^{W}}$) agree quantitatively with the calculated band gap variation in hBLs with AA and AB stacking. However, we noted that A-exciton peak in WSe_2 ($\mathrm{X_A^{W}}$) exhibits a significantly larger energy shift. The neutral and charged excitons can be distinguished in PL spectra. In **Supplementary Figure 7b**, we also include the energy of neutral A exciton determined from PL measurements. The energy variation agrees very well with the calculated bandgap energy, indicating that the $\mathrm{X_A^{W}}$ peak measured by $\Delta R/R$ is dominated by trion absorption.

Supplementary Note 5. Theoretical analysis of interlayer dipole

Here we consider lattice-matched $MoSe_2/WSe_2$ commensurate heterobilayers with AA and AB stacking (without interlayer twist). The Bloch function of the conduction (valence) band edge at τ'K (τK) valley consists predominantly of the d_{z^2} ($d_{x^2-y^2} + i\tau d_{xy}$) orbitals with magnetic quantum $m = 0$ ($m = 2\tau$) on Mo (W) sites in the $MoSe_2$ (WSe_2) layer, and can be written as

$$\psi_{c,\tau'\mathbf{K}}^{\mathrm{Mo}}(\mathbf{r}) \cong \sum_{n'} e^{i\tau'\mathbf{K}\cdot\mathbf{R}_{n'}} d_{m=0}^{\mathrm{Mo}}(\mathbf{r}-\mathbf{R}_{n'}) \,, \tag{1}$$

$$\psi_{v,\tau\mathbf{K}}^{\mathrm{W}}(\mathbf{r}) \cong \sum_{n} e^{i\tau\mathbf{K}\cdot\mathbf{R}_{n}} d_{m=2\tau}^{\mathrm{W}}(\mathbf{r}-\mathbf{R}_{n}) \,, \tag{2}$$

where $d_{m=0}^{\mathrm{Mo}}(\mathbf{r}-\mathbf{R}_{n'})$ and $d_{m=2\tau}^{\mathrm{W}}(\mathbf{r}-\mathbf{R}_{n})$ are the orbitals of Mo and W atoms; $\mathbf{R}_{n'}$ ($\mathbf{R}_n$) is the location of the n'-th (n-th) Mo (W) atom in the $MoSe_2$ (WSe_2) layer. The interlayer transition dipole of $\mathrm{X}_{\tau'\tau}^{\mathrm{I}}$ can be expressed as

$$\mathbf{D}_{\tau'\tau}(\mathbf{r}) \cong \langle \psi_{v,\tau\mathbf{K}}^{\mathrm{W}}(\mathbf{r}) | \hat{\mathbf{D}} | \psi_{c,\tau'\mathbf{K}}^{\mathrm{Mo}}(\mathbf{r}) \rangle$$

$$= \sum_{n,n'} e^{i(\tau'\mathbf{K}\cdot\mathbf{R}_{n'} - \tau\mathbf{K}\cdot\mathbf{R}_{n})} \langle d_{m=2\tau}^{\mathrm{W}}(\mathbf{r}-\mathbf{R}_{n}) | \hat{\mathbf{D}} | d_{m=0}^{\mathrm{Mo}}(\mathbf{r}-\mathbf{R}_{n'}) \rangle \,. \tag{3}$$

At each Mo site, $\mathbf{r} = \mathbf{R}_{n'}$, we have

$$\mathbf{D}_{\tau'\tau}(\mathbf{R}_{n'}) = \sum_{n} e^{i(\tau'\mathbf{K}\cdot\mathbf{R}_{n'} - \tau\mathbf{K}\cdot\mathbf{R}_{n})} \langle d_{m=2\tau}^{\mathrm{W}}(\mathbf{R}_{n'}-\mathbf{R}_{n}) | \hat{\mathbf{D}} | d_{m=0}^{\mathrm{Mo}}(0) \rangle \,. \tag{4}$$

Then we define $\mathbf{r}_n \equiv \mathbf{R}_{n'} - \mathbf{R}_n$, i.e., the vector connecting all W sites with a given Mo site, and set $\mathbf{R}_{n'} = 0$. Therefore, the interlayer transition dipole at each Mo site is

$$\mathbf{D}_{\tau'\tau}(0) = \sum_{n} e^{-i\tau\mathbf{K}\cdot\mathbf{r}_{n}} \langle d_{m=2\tau}^{\mathrm{W}}(\mathbf{r}_{n}) | \hat{\mathbf{D}} | d_{m=0}^{\mathrm{Mo}}(0) \rangle \,, \tag{5}$$

which connects the orbitals on Mo and W sites in different layers [1]. Since the orbitals of W and Mo atoms are well localized around $\mathbf{R}_n$ and $\mathbf{R}_{n'}$, the interlayer transition dipole is dominated by the nearest-neighbor orbitals of W and Mo atoms. If we consider the nearest-neighbor dipoles between Mo and W orbitals as a first approximation, the total transition dipole is given by

$$\mathbf{D}_{\tau'\tau} \propto \sum_{n=1,2,3} e^{-i\tau\mathbf{K}\cdot\mathbf{r}_{n}} \langle d_{m=2\tau}^{\mathrm{W}}(\mathbf{r}_{n}) | \hat{\mathbf{D}} | d_{m=0}^{\mathrm{Mo}}(0) \rangle \,, \tag{6}$$

which is the superposition of the three dipoles associated with a distinct phase factor $e^{i\tau\mathbf{K}\cdot\mathbf{r}_n}$. According to the theory proposed by Yu *et al.* [1], the interlayer transition dipole also acquires contributions from coupling to intralayer excitons via interlayer hopping. Based on the symmetry analysis detailed in Yu *et al.* [1], the σ^+ and σ^- components of $\mathbf{D}_{\tau'\tau}$ are [1]:

$$\mathbf{e}_{\tau} \cdot \mathbf{D}_{\tau'\tau} \propto e^{-i\tau\mathbf{K}\cdot\mathbf{r}_1} + e^{-i\tau\mathbf{K}\cdot\mathbf{r}_2} + e^{-i\tau\mathbf{K}\cdot\mathbf{r}_3}\,, \tag{7}$$

$$\mathbf{e}_{-\tau} \cdot \mathbf{D}_{\tau'\tau} \propto e^{-i\tau\mathbf{K}\cdot\mathbf{r}_1} + e^{-i\tau\left(\mathbf{K}\cdot\mathbf{r}_2+\frac{2\pi}{3}\right)} + e^{-i\tau\left(\mathbf{K}\cdot\mathbf{r}_3+\frac{4\pi}{3}\right)}\,, \tag{8}$$

where $\mathbf{e}_{\pm} = (\mathbf{x} \pm i\mathbf{y})/\sqrt{2}$ is the unit vector of $\sigma^{\pm}$ polarization. According to the valley optical selection rule, the corresponding optical helicity for the possible valley configurations of bright X^{I} states in AA and AB stacking are listed in **Supplementary Table 1** and schematically shown in **Supplementary Figure 9**. For the analysis of interlayer dipole strength and polarization ellipticity in lattice-matched $MoSe_2$/WSe_2 hBL with an arbitrary interlayer translation, see Yu *et al.* [1] for details.

Supplementary Note 6. Interlayer spin-valley transfer

We performed optical-pump-induced circular dichroism (CD) spectroscopy to study the interlayer spin-valley transfer processes. The experimental setup is shown in **Supplementary Figure 10**.

According to Schaibley *et al.* [2], the spin and valley polarized carriers created by the circularly-polarized pump beam have two dominant effects on the probe beam: (1) band filling effect for co-polarized probe and (2) trion formation for cross-polarized probe, as illustrated schematically in **Supplementary Figure 11**. Consider the case of σ^+ pump injecting +K polarized electrons in the $MoSe_2$ layer, the exciton absorption measured by σ^+ probe will be partially blocked and blue shifted due to the band filling effect (**Supplementary Figure 11a,b**). For σ^- probe at the opposite –K valley, the dominant effect is trion (X^-) formation, leading to increased trion absorption and reduced neutral exciton absorption (**Supplementary Figure 11c,d**). The difference between the σ^+ and σ^- probes thus gives rise to the CD response, resulting in a CD line shape as shown in **Supplementary Figure 11e**, where the peak and dip are close to the exciton and trion resonances, respectively. For the case of injecting +K polarized holes in the WSe_2 layer using σ^+ pump, the CD response also arises from the band filling effect and the trion (X^+) formation (**Supplementary Figure 11f, g**), which results in a similar CD line shape.

In **Supplementary Figure 12**, we show the CD spectrum measured from monolayer WSe_2 regions using σ^+ pump at 1.797 eV (690 nm). The observed CD spectrum is consistent with the

line shape feature shown in **Supplementary Figure 11**. By using two Lorentzian functions to fit the CD line shape, we obtain a peak and a dip, which are close to the exciton and trion resonances of monolayer WSe_2 measured by the differential reflectance. Since our CVD grown WSe_2 is weakly p-type, the trion peak is attributed to the formation of positively charged excitons (X^+).

Now we discuss the CD response in the $WSe_2/MoSe_2$ hBLs. In **Supplementary Figure 13a,b**, we show the CD spectra for AA- and AB-stacked hBLs using above-gap σ^+ excitation at 1.797 eV. The above-gap excitations create +K polarized carriers in both WSe_2 and $MoSe_2$ layers. After interlayer charge transfers, electrons (holes) are localized in the $MoSe_2$ (WSe_2) layer. We observed CD responses near the exciton resonances of both $MoSe_2$ and WSe_2. We used two positive (blue) and two negative (orange) Lorentzian functions to fit the CD spectra. The positive (negative) peaks corresponds well with the exciton (trion) peaks X^W (T^W) and X^{Mo} (T^{Mo}) of WSe_2 and $MoSe_2$ measured by the differential reflectance and PL spectra. The spectral features of the CD responses thus confirm the picture of creating +K-polarized electrons (holes) in $MoSe_2$ (WSe_2) layer by the above-gap σ^+ excitation.

To gain insight on the interlayer spin-valley transfer, we used σ^+ pump at $MoSe_2$ and probe the CD response of WSe_2 (**Supplementary Figure 13c,d**), which reflects the spin polarized holes transferred from the +K valleys of $MoSe_2$ to the +K valley of WSe_2. We found that the CD line shapes of WSe_2 using σ^+ pump at $MoSe_2$ exciton energy (1.642 eV) are almost identical with that using above-gap σ^+ excitation at 1.797 eV for both stacking. This result clearly demonstrates that the interlayer hole transfer is a spin-conserving process, regardless of the stacking orientation. We have also measured the CD response near the $MoSe_2$ exciton resonance using σ^+ pump at the $MoSe_2$ layer (**Supplementary Figure 13c,d**). In the experiment, the excitation energy was increased slightly to 1.664 eV in order to reject the pump beam from the measurements. The CD line shape of $MoSe_2$ using σ^+ pump at the $MoSe_2$ layer are also similar to that using above-gap excitation, indicating the generation of +K polarized electrons in the $MoSe_2$ layer. Furthermore, we observed enhanced CD responses of $MoSe_2$ by a factor of 3-4 when pumping at the $MoSe_2$ layer, in comparison with that using above-gap excitations. This suggests that using resonant (near resonant) excitation at the $MoSe_2$ exciton energy can significantly increase the electron valley polarization in $MoSe_2$, in consistent with the enhanced P_C of interlayer excitons shown in **Fig. 3b**.

Supplementary Note 7. Formation of bright X^I states in AB stacked hBLs

According to the valley optical selection rule for AB stacking and the sign of measured P_C shown in **Fig. 4b**, we determine the preferential valley configuration of bright X^I state as X^I_{-+} when using σ^+ excitation at the $MoSe_2$ layer and X^I_{+-} when using σ^+ excitation at the WSe_2 layer. According to Schaibley *et al.* [2], the interlayer charge transfer process is dominated by a spin-conserving transfer to the lowest energy band, independent of the interlayer momentum mismatch. Our CD measurements (see **Supplementary Note 6)** also support this picture. Therefore, using σ^+ excitation at $MoSe_2$ layer, a majority of dark interlayer state X^I_{++} is created in the steady state. The formation of bright X^I_{-+} state may meditated through a spin-conserving intervalley scattering in the $MoSe_2$ layer (**Supplementary Figure 14 a**). When using σ^+ excitation at the +K valley of WSe_2 layer, the spin-conserving interlayer charge transfer also implies to form a majority of dark X^I_{++} in steady state. However, the formation of bright X^I_{+-} state requires spin flip processes (**Supplementary Figure 14 b**), which is expected to be energetically unfavorable. Nevertheless, the low circular polarization for X^I in AB stacked hBL suggest that there is a competing channel for the formation of X^I_{-+} states. Further studies, such as using time-resolved Kerr rotation spectroscopy, is necessary to clarify which path is the dominant process.

The existence of lower-lying dark state in AB stacked hBLs will quench the emission from bright X^I states, particularly at low temperatures. Therefore, the PL emissions from AB stacked hBLs are less efficient than those from hBLs with AA stacking. As shown in **Supplementary Figure 15**, the PL intensity for the investigated AB stacked hBLs is generally weaker by a factor of 2-5 in comparison with those of AA stacking, which may imply the presence of lower-lying dark state in AB stacked hBLs.

Supplementary References:

1. Yu, H., Wang, Y., Tong, Q., Xu, X. & Yao, W. Anomalous light cones and valley optical selection rules of interlayer excitons in twisted heterobilayers, *Phys. Rev. Lett.* **115**, 187002 (2015).
2. Schaibley, J. R. *et al.* Directional interlayer spin-valley transfer in two-dimensional heterostructures, *Nat. Commun.* **7**,13747 (2016).
